# Inadequacy of Body Weight-Based Recommendations for Individual Protein Intake—Lessons from Body Composition Analysis

**DOI:** 10.3390/nu9010023

**Published:** 2016-12-31

**Authors:** Corinna Geisler, Carla M. Prado, Manfred J. Müller

**Affiliations:** 1 Institut für Humanernährung und Lebensmittelkunde, Christian-Albrechts-Universität zu Kiel, Düsternbrooker Weg 17, D-24105 Kiel, Germany; mmueller@nutrfoodsc.uni-kiel.de; 2 Alberta Institute for Human Nutrition, Department of Agricultural, Food and Nutritional Science, University of Alberta, 4-002 Li Ka Shing Centre, Edmonton, AB T6G 2P5, Canada; carla.prado@ualberta.ca

**Keywords:** lean mass, healthy and clinical populations, Caucasian, protein needs, protein intake-body composition relationship

## Abstract

Current body weight-based protein recommendations are ignoring the large variability in body composition, particularly lean mass (LM), which drives protein requirements. We explored and highlighted the inter-individual variability of weight versus body composition-adjusted protein intakes by secondary analysis in three cohorts of (1) 574 healthy adults (mean ± SD age: 41.4 ± 15.2 years); (2) 403 cirrhotic patients (age: 44.7 ± 12.3 years) and (3) 547 patients with lung cancer (age: 61.3 ± 8.2 years). LM was assessed using different devices (magnetic resonance imaging, dual-energy X-ray absorptiometry, computer tomography, total body potassium and bioelectrical impedance), body weight-based protein intake, its ratio (per kg LM) and mean protein requirement were calculated. Variability in protein intake in all cohorts ranged from 0.83 to 1.77 g protein per kg LM per day using (theoretical protein intake of 60 g protein per day). Calculated mean protein requirement was 1.63 g protein per kg LM per day; consequently, 95.3% of healthy subjects, 100% of cirrhotic and 97.4% of cancer patients would present with a low protein intake per kg LM. Weight-adjusted recommendations are inadequate to address the LM specific differences in protein needs of healthy subjects or clinical populations. Absolute protein intake seems to be more relevant compared to the relative proportion of protein, which in turn changes with different energy needs.

## 1. Introduction

Current guidelines for protein intake include the estimated average requirement (EAR) and the recommended dietary allowance (RDA), which are, respectively, 0.66 and 0.80 g per kg body weight per day [1]. These recommendations are independent of age, sex and body composition. The Institute of Medicine’s Food and Nutrition Board also defined protein needs as Acceptable Macronutrient Distribution Range (AMDR), suggesting a range versus a constant amount of protein intake. This range was based on 10% to 35% of energy intake, with a lower limit corresponding to the RDA and an upper limit up to 3.0 g protein per kg body weight per day. This wide range is not specific as an optimal value is not provided and optimized by age, sex and body composition. Additionally, inter-individual variances in protein intake based on body weight or energy adjusted protein recommendations may explain opposing results of the relationship between nutrient intake and body composition observed in previous studies. Houston et al. [2] showed in the Health Aging and Body Composition study that energy adjusted protein intake was positively associated with having a higher lean mass after three years. Participants in the highest protein intake (1.2 g per kg body weight per day) quintile lost less than 40% of lean mass (LM) when compared with participants in the lowest quintile (0.7 g per kg body weight per day) [2]. In contrast, Campbell et al. [3] found that meeting the RDA of protein intake (0.8 g per kg body weight per day) during a controlled eucaloric diet resulted in loss of leg lean mass with no change in body weight. In a previous work, Argilés et al. [4] showed that disturbances in muscle protein metabolism (degradation and impaired synthesis) were related to sarcopenia. It is important to note that aging might lead to lower muscle protein synthesis compared to healthy young adults [5]. Other authors also showed that the RDA of protein failed to prevent muscle loss in sarcopenic subjects [6,7].

These data may favor the idea that protein intake should be based on body composition (e.g., LM) rather than body weight, which may reflect true protein needs of individuals [8]. Likewise, the observed inter-individual variance of body composition in healthy and in poor health subjects additionally highlights the inadequacy of weight-adjusted recommendations, i.e., they do not meet the LM derived protein needs [9,10]. Protein recommendations are generally based on body weight. Both over- and undernutrition could be present in healthy subjects and various clinical populations since body composition (LM) may vary greatly. In liver cirrhosis, body weight is nearly normal in many patients, whereas in cancer patients the prevalence of sarcopenia and sarcopenic obesity in normal and overweight patients could be up to 20% and more [11]. Nonetheless, the role of body composition in terms of inter-individual variance in protein intake has not been investigated in a population of healthy subjects or in various clinical patients. Therefore, three different unrelated populations (healthy and various clinical) were used to show these differences. Here, we explore this concept, demonstrating the inter-individual variability of weight versus body composition adjusted protein intakes.

## 2. Materials and Methods

### 2.1. Study Populations

#### 2.1.1. Healthy Subjects

Data from 574 healthy adult Caucasians (55.6% females and 44.4% males) varying in age (18–82 years) and body mass index (BMI) (16.8–46.7 kg/m^2^) recruited as previous participants of body composition studies were used. Age and BMI were not normally distributed in the whole study group (Kolmogorov–Smirnov test *p* < 0.001). Healthy subjects were recruited from the local community (Kiel City and surroundings) by advertisements in newspapers and notice board postings. Exclusion criteria included acute or chronic diseases, regular medication, smoking, metallic implants or artificial joints depending on the study protocol. Body composition analysis took place at the German Reference Center for Body Composition at the Institute of Human Nutrition [12,13,14].

In addition, data from 32 healthy young males aged 20–37 years with a BMI ranged between 20.7 to 29.3 kg/m^2^ who attended a caloric restriction-overfeeding study were used for secondary data analysis [15,16]. Male subjects underwent a protocol with three different stages of overfeeding (OF), refeeding (RF) and caloric restriction (CR). Among other parameters changes in body weight, lean mass and fat mass as well as nitrogen balance were assessed. Protein intake was calculated as 15% of energy. Energy intakes were 1353 ± 154 kcal per day (CR) and 4059 ± 452 kcal per day (OF, RF) during caloric restriction (−50% of energy need) and overfeeding (+50% of energy need), respectively. Protein intakes were 49 ± 6 g and 146 ± 17 g per kg body weight per day, respectively, during CR and OF as well as RF. The detailed study design, protocol and results were described previously [15,16].

The studies followed the guidelines of the Declaration of Helsinki; all procedures involving human subjects were approved by the local ethical committee of the Christian-Albrechts-Universität zu Kiel (last approved version A100/13A; 2014). Written informed consent was obtained from all subjects before participation.

#### 2.1.2. Patients with Liver Cirrhosis

Patient data (collected between 1986 and 1993) were obtained from a previous study at the Medizinische Hochschule Hannover’s liver transplantation program [17]. The Child-Pugh Score (CHILD) characterizes deterioration of the hepatocellular function and its consequences in patients with liver cirrhosis. The score is used to determine the prognosis, required strength of treatment and need of liver transplantation in cirrhotic patients. The Child-Pugh Score is defined by serum bilirubin (Child-Pugh Score A <2.0 mg/dL; Child-Pugh Score B 2.0–3.0 mg/dL); Child-Pugh Score C >3.0 mg/dL), serum albumin (>3.5 g/dL; 3.0–3.5 g/dL; <3.0 g/dL), ascitis (none; easily controlled; poorly controlled), neurological disorders (none; minimal; advanced coma) and nutrition (excellent; good; poor), whereas Child-Pugh Score A showed the best prognosis. Baseline data of 403 patients with biopsy-proven liver cirrhosis with Child-Pugh Score A (34.2%), B (52.4%) and C (13.4%) were used for secondary data analysis. 

#### 2.1.3. Cancer Patients

Baseline data from a prospective intervention study were used [18]. A total of 547 patients (~71% males) with stage III or IV non-small lung cancer and available computerized tomography (CT) scans were used. Patients receiving megestrol acetate, testosterone or other like agents for unintended weight loss were not included. This study was approved by the Florida State University Institutional Review Board.

### 2.2. Body Composition Analysis in Study Populations

#### 2.2.1. Healthy Subjects

##### Magnetic Resonance Imaging (MRI)

Skeletal muscle mass (MM) was measured using whole body multislice MRI. Scans were obtained with a 1.5T scanner (Magnetom Vision Siemens, Erlangen, Germany) using a T1 weighted gradient-echo sequence. Images were analyzed from wrist to ankle using the SliceOmatic software (version 4.3; Tomovision, Montreal, QC, Canada). MRI estimated muscle volumes were converted to mass using a density of 1.04 kg/L [19]. Intra-observer coefficient of variation was 1.8 % for total MM.

##### Dual-Energy X-ray Absorptiometry (DXA)

In healthy subjects, whole body scans were performed using DXA (Hologic Discovery A densitometer; whole-body-software 12.6.1:3; Hologic, Inc., Bedford, MA, USA). Whole body and regional (arms and legs) lean soft tissue (LST; bone not included) were differentiated by specific anatomical landmarks [20] using manually adjusted computer-generated default lines. Appendicular skeletal muscle mass index (ASMI) and fat mass index (FMI) were calculated by mass (kg) per height per m^2^.

##### Quantitative Magnetic Resonance Imaging (QMR)

QMR was used for daily measurements of fat free mass (FFM; weight minus fat mass) during the caloric restriction study in healthy young males (EchoMRI-AH; Echo Medical Systems, Houston, TX, USA). QMR measurements have a good precision and validity with a low standard deviation (0.133 kg to 0.221 kg) and coefficient of variation (0.44% to 0.69%) [21,22]. 

#### 2.2.2. Patients with Liver Cirrhosis

##### Bioelectrical Impedance (BIA)

Cirrhotic patients had a tetra polar BIA measurement of resistance (R) and reactance (Xc) taken between the right wrist and ankle during supine position with an operating frequency of 50 kHz (Nutri Guard M Analyzer; Data Input, Darmstadt, Germany). Fat free mass (FFM) was calculated using the prediction equation of Sun et al. [23]. Fat mass (FM) was derived from the equation:

FM = body weight − FFM(1)

Indices of FFM (FFMI) as well as FM (FMI) were calculated by mass (kg) per height per m^2^.

##### Total Body Potassium (TBK)

TBK was measured by counting 40K with a whole body counter and was described previously [17] with a precision of >97%. Counted TBK was converted into mmol and total body protein (TBPro_TBK_) was calculated using the modified equations of Ellis et al. [24].

#### 2.2.3. Cancer Patients

##### Computer Tomography (CT)

In cancer patients, CT images were taken for body composition analysis. CT images at the third lumbar vertebrae were analyzed using Slice-O-matic software V. 4.3 (Tomovision, Montreal, QC, Canada). Pre-established thresholds of Hounsfield units were used to identify total muscle cross-sectional area as described in detail previously [9]. An extrapolation of cross-sectional area to LST (bone not included) was employed using the equation described in Mourtzakis et al. [25]. Appendicular skeletal muscle mass index (ASMI) and fat mass index (FMI) were calculated by mass (kg) per height per m^2^.

##### Standardization of Lean Mass 

In order to standardize the terms LST (DXA, CT), MM (CT, MRI) and FFM (QMR and BIA) using different study populations, the unified term “lean mass (LM)” was chosen. Although techniques are assessing different compartments [8,26], we will use the terms interchangeably for the purpose of our presentation.

##### Definition of Sarcopenia

In healthy subjects sarcopenia was defined according to individual BMI, age and sex-specific cut-offs for body-composition phenotypes based on ASMI and FMI [27] assessed by DXA. ASMI and FMI were combined to define non-sarcopenic and sarcopenic phenotypes.

In patients with liver cirrhosis, the criteria of the European Society of Clinical Nutrition and Metabolism (ESPEN) [27] were used. These guidelines defined low lean mass as FFM index (FFMI) <15 kg/m^2^ and <17 kg/m^2^ in females and males, respectively [27]. 

Cancer patients were classified as sarcopenic according to Prado et al. [28] cut-off points of CT muscle cross-sectional area at L3 (cm^2^/m^2^). Corresponding cut-off points were <38.5 cm^2^/m^2^ in females and <52.4 cm^2^/m^2^ in males. 

Additionally, MM and TBPro_TBK_ data were used in healthy subjects and cirrhotic patients to define sarcopenic phenotypes. Sarcopenia was defined using the cut points of the European Working Group on Sarcopenia in Older People (EWGSOP) for severe sarcopenia based on absolute muscle mass divided by height in square meter in healthy females (<5.75 kg/m^2^) and males (<8.50 kg/m^2^) [29] and TBPro_TBK_ < 6.2 kg (lowest 10% of healthy persons) in cirrhotic patients.

### 2.3. Assessment of Resting Energy Expenditure (REE) in Study Populations

#### 2.3.1. Healthy Subjects

Indirect calorimetry (REE) was performed using a ventilated-hood system (Vmax spectra 29n; SensorMedics BV, Viasys Healthcare, Bilthoven, The Netherlands).The procedure was described in detail elsewhere [12,13,14]. 

#### 2.3.2. Patients with Liver Cirrhosis

Indirect calorimetry (REE) was performed using a ventilated-hood system (Deltatrac Metabolic Monitor; Datex-Instrumentarium, Helsinki, Finland) the procedure was described in detail elsewhere [17]. 

#### 2.3.3. Cancer Patients

REE was calculated by the equation of Harris and Benedict [30]. 

### 2.4. Calculation of Total Energy Expenditure (TEE)

REE was multiplied by an activity level of 1.55 to calculate total energy expenditure (TEE) as a measure of total energy needs. Energy-based protein intake was calculated as 15% of TEE.

#### Statistical Analysis

SPSS 22.0 (SPSS Inc., Chicago, IL, USA) was used for analyses with significance level set at *p* < 0.05; independent *t*-test was used to calculate differences between females and males. Paired *t*-test was used to compare differences between measured and calculated REE in healthy subjects and cirrhotic patients (Table S1). Total protein requirement was calculated on a RDA of 0.8 g protein per kg body weight per day for healthy subjects and clinical populations [1]. Calculated protein requirement was divided by LM to generate a ratio of protein intake in grams per kg LM. Regression analysis was used to test the relationship between calculated absolute protein intake (g protein per day) and protein intake per LM (g protein per kg LM per day). 

### 2.5. Calculations of Theoretical Protein Requirements

Nitrogen balance data from the caloric restriction study (see methods and materials) were used to calculate the relationship of nitrogen to protein balance. The aim was to evaluate corresponding cut-offs of protein/body weight or protein/LM where nitrogen balance would be zero (intersection point on *x*-axis). Linear regression analyses were used to calculate these cut-offs. The following equations were converted to “*x*” in order to calculate the corresponding cut-offs:

*y = 10.13x − 9.881* (*R*^2^ = 0.07; *p* < 0.05) (2)

*y = 5.662x − 8.783* (*R*^2^ = 0.07; *p* < 0.05) (3)

where *y* (nitrogen balance) is equal to zero and *x* represented g protein per kg body weight per day or g protein per kg LM per day. 

The result of g protein per kg LM per day (=*x*) was obtained from LM based on FFM assessment (see methods and materials section). Thus, the result was converted with Equation (4):

g protein per kg LM per day = *1.055x − 0.009* (*R*^2^ = 0.07; *p* < 0.05) with LM based on DXA (4)

These protein requirement cut-offs were used to calculate the prevalence of participants who were above or below these protein requirements. Afterwards, information regarding the prevalence would be used to calculate and identify the necessary amount of protein to define an adequate supply. 

## 3. Results

### 3.1. Healthy and Clinical Populations Characteristics

Descriptive characteristics of healthy subjects, patients with liver cirrhosis or cancer are shown in Table 1. Mean age of patients with liver cirrhosis was comparable to healthy subjects, whereas cancer patients had a higher mean age (*p* < 0.05). The prevalence of sarcopenia was 19.0% in healthy subjects, 29.3% in cirrhotic and 46.6% in cancer patients. Using the alternative EWGSOP cut points 7.1% of the healthy subjects and 17.9% of cirrhotic patients (TBPro_TBK_ < 6.2 kg) would be defined as sarcopenic. Muscle mass_MRI_ and LM_DXA_ correlated significantly (*r* = 0.95; *p* < 0.05) in healthy subjects as well as LM_BIA_ and TBPro_TBK_(*r* = 0.68; *p* < 0.05) in cirrhotic patients. 

**Figure 1:**
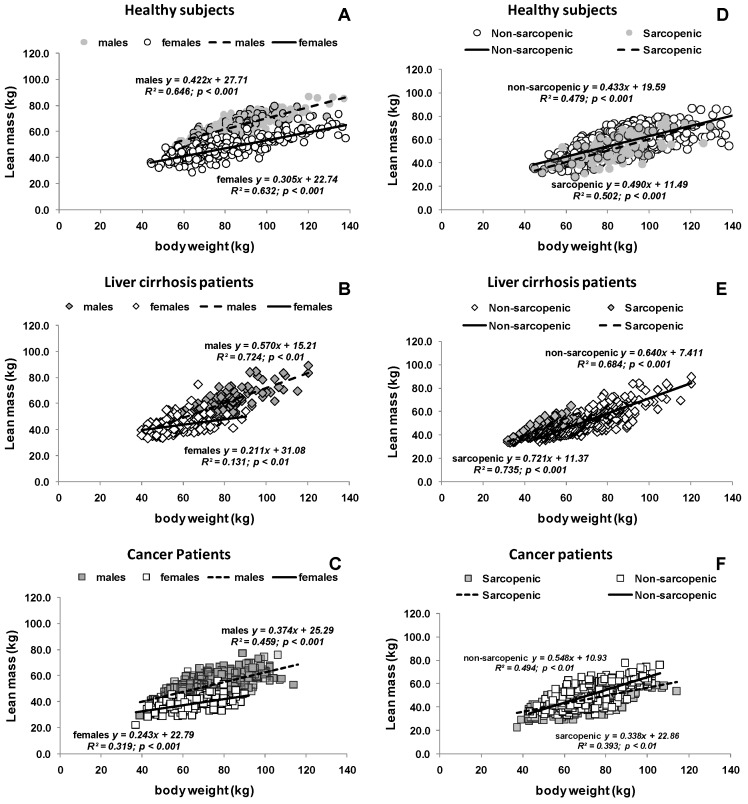
Relationship between lean mass and body weight in healthy subjects, patients with liver cirrhosis and cancer. The relationship is presented stratified by: sex (**A**–**C**); and sarcopenic phenotype (**D**–**F**). Lean mass was measured as LST by DXA or CT in healthy subjects and cancer patients and FFM by BIA in cirrhotic patients (solid line and broken line show the corresponding regression line).

Positive associations were observed between body weight and lean mass in all cohorts. Lean mass increased with increasing body weight (Figure 1A–C). This was also true in non-sarcopenic and sarcopenic individuals from all cohorts, although a weaker association was observed in patients with cancer (Figure 1D–F).

### 3.2. Estimated Protein Intake Based on either Body Weight or Body Composition

**Figure 2:**
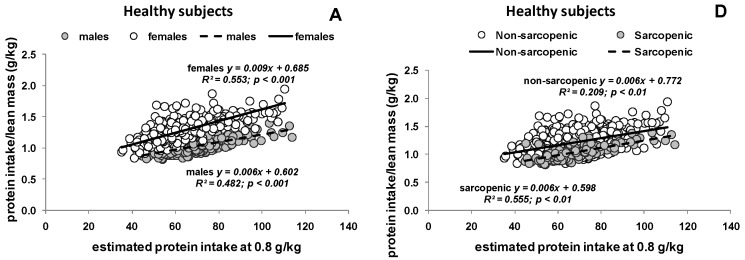

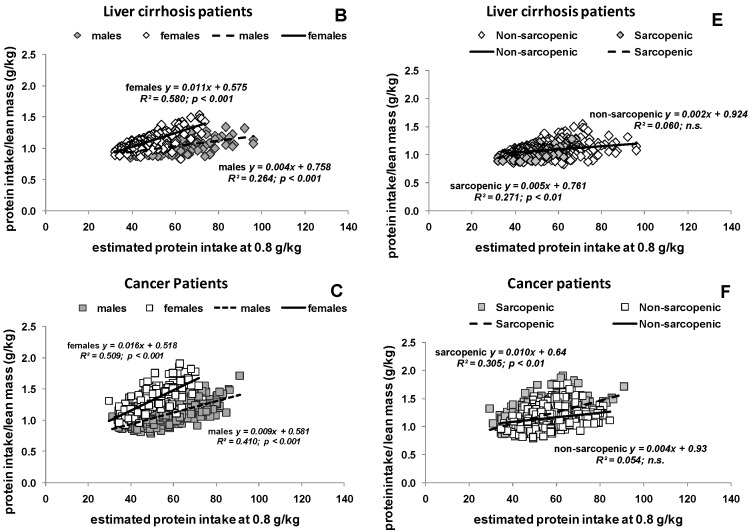
Relationship between estimated (body weight based) protein intake at 0.8 g protein per kilogram body weight (g/kg) and the ratio of protein intake (g) divided by lean mass (kg) in healthy subjects, patients with liver cirrhosis and cancer. The relationship is shown stratified by: sex (**A**–**C**); and sarcopenic phenotypes (**D**–**F**) (solid line and broken line show the corresponding regression line). Lean mass was measured as lean soft tissue by DXA in healthy subjects, fat free mass by bioelectrical impedance in cirrhotic patients and by computer tomography in cancer patients.

Figure 2 shows the relationship between protein intake as 0.8 g per kg body weight per day and the corresponding protein intake expressed per kg LM per day. There was an effect of sex in healthy subjects and patients (Figure 2A–C), which is explained by sex differences in lean mass. Variability in protein intake per lean mass in all cohorts ranged from 0.83 g to 1.77 g protein per kg LM per day (sarcopenic 0.99 g to 1.77 g and non-sarcopenic 0.83 g to 1.50 g) based on a suggested protein intake of 60 g protein per day. 

When protein intake was calculated based on 15% of TEE, average mean protein intake was significantly higher than protein intake estimated at 0.8 g per kg body weight per day (94.8 ± 18.4 vs. 66.0 ± 15.1 g per day; *p* < 0.001). When expressed per kg LM, protein intake ranged from 1.26 g to 2.48 g protein per kg LM per day.

### 3.3. Potential Consequences of Variable Protein and Energy Recommendations

Data from a caloric restriction-overfeeding experiment in young male subjects [16] showed a nitrogen balance of −3.6 ± 3.5 g and 9.0 ± 6.6 g per day during CR and OF, respectively. Nitrogen balance data during CR were related to g protein per kg LM per day or g per kg body weight per day. Linear regression analysis resulted in protein requirements of 0.97 g per kg body weight per day and 1.55 g protein per kg LM per day for an isnitrogenous status. Converting previous result of 1.55 g protein kg LM per day by Equation (4) (see Section 2.5) resulted in 1.63 g protein per kg LM per day. 

When comparing the calculated protein per kg LM for each subject with these theoretical protein needs, 95.3% of healthy subjects, 100% of cirrhotic and 97.4% of cancer patients are below these protein recommendation. Using protein intake as 15% of TEE, 28.4% of healthy subjects, 3.7% of cirrhotic and 36.9% of cancer patients would present a low protein intake. All healthy individuals would meet the new cut-off for protein intake with an amount of 1.5 g per kg body weight per day (equivalent to 17.5% of TEE). In cirrhosis and cancer patients, individuals would reach the new cut-off with a recommendation of 1.5 and 1.6 g per kg body weight per day (equivalent to 15% or 21% of TEE), respectively.

Considering the sarcopenic phenotype, 1.25% (body weight based protein intake) and 17.2% (protein intake 15% of TEE) of all subjects who consumed >1.63 g protein per kg LM per day were sarcopenic.

## 4. Discussion

The huge variability in body composition resulted in considerable variances in “effective” protein intakes (protein intakes considering LM), when compared with our usual adjustment per kilogram body weight. Our results indicated that current protein intake recommendations ignore the large variability of body composition phenotypes. We described a wide range of “effective” protein intake per kg LM (0.83 to 1.77 g protein per kg LM per day), which is in line with findings from Prado et al. [9] and Rafii et al. [31] who calculated a variability in protein intake per kg LM of 1.62 ± 0.14 g per kg LM per day in older women, compared with 1.14 ± 0.09 g per kg LM per day in younger counterparts. Similar results were described by Campbell et al. [32], according to age related changes of LM (DXA). Thus, varying differences in phenotypes of healthy and morbid individuals could account for higher protein needs per LM.

A minor percentage (<5.0%) of healthy subjects were consuming more than 1.63 g protein per kg LM per day if the general RDA of 0.8 g protein per kg body weight per day was used to calculate protein intake and 37.0% of them were sarcopenic. Furthermore, 68.7% of healthy subjects were above the new cut-off if protein intake was energy-based (15% of TEE) and nearly one-fifth of them were sarcopenic. This would represent an “overfeeding” with protein per LM in sarcopenic or sarcopenic obese subjects, whereas several differences in metabolism of healthy subjects and patients (e.g., disturbances in muscle protein metabolism, linked substrate metabolism) could in fact increase protein needs [4].

Considering the high-risk sarcopenic phenotype, just 1.25% to 17.2% of all sarcopenic subjects were beyond our detected protein cut-off of 1.63 g protein per kg LM per day. In a theoretical exercise, Prado et al. [28] showed how the variability in body composition could increase risk of adverse outcomes during chemotherapy. Here, we highlighted that the same variability can influence protein needs, which are driven by LM. Protein intake per LM was very variable in healthy subjects as well as in patients with cirrhosis or cancer. This implies that an individual may reach recommended protein intake (RDA), while another may not depending on his/her body composition phenotype (e.g., normal, sarcopenic, sarcopenic obese or cachectic). These findings might explain the results from previous studies with different evidence of age-related muscle protein synthesis [33,34]. In a 14-week controlled feeding study, older women and men consuming an eucaloric diet providing the RDA of protein [35], showed a decrease in LM and muscle midtight area over time, suggesting an inadequate RDA value for protein in elderly. Likewise, Houston et al. [2] showed that older adults who consumed the lowest amount of protein (0.7 g protein kg body weight per day) lost more LM during a three-year period than a person consuming higher amounts of protein. 

An additional consideration of potential impact would be meeting protein recommendations for individuals who have a sarcopenic obese phenotype. Dietary intervention for these individuals ought to provide an adequate protein intake per LM to prevent loss of LM [36], while guaranteeing a low energy intake for weight loss [37]. The same would be important in clinical scenarios, where an adequate supply of energy and protein is essential to prevent loss of LM, while optimizing energy intake and hence, overall body weight (and fat mass). As such, absolute protein intake seems to be more important than protein intake expressed as a percentage of TEE [38] to prevent LM loss and to ensure that individuals are not in a negative protein balance. Morais et al. [39] showed in his review, that nitrogen balance studies indicate a higher protein (1.0–1.3 g protein per kg body weight per day) requirement to maintain nitrogen balance in healthy elderly. He said that this could be explained by their lower energy intake, which is in line with the findings of Bunker et al. [40] who compared daily energy intake, excretion and retention of nitrogen in healthy and housebound women and men. Both groups consumed the same percentage of energy from protein (≈14%) but housebound participants were in a negative N balance (−95 mmol per day) when compared with healthy ones [40]. Müller et al. [16] determined daily nitrogen data of young males during a caloric restriction study to control protein balance during under- and overfeeding. Results of this study showed that a protein intake at 15% of TEE during CR (−50% energy) and RF (+50% energy) was associated with a higher loss (CR) and lower regain (RF) of LM [16]. Our results showed that calculated resting energy expenditure (REE according to Harris and Benedict) was higher than the actual measured resting energy expenditure (by indirect calorimetry) in healthy subjects and cirrhotic patients (Table S1). Thus, an adequate or higher energy intake could be associated with an inadequate intake of protein in relation to energy. Consequently, individuals may present with sarcopenic obesity, therefore with lower energy but higher or elevated protein needs. This could be important for nutrition recommendations in populations where the prevalence of sarcopenia and sarcopenic obesity is high, such as in cancer, for example. Anandavadivelan et al. [11] showed a prevalence of 43.0% and 14.0% of sarcopenic and sarcopenic obese, respectively, in 72 patients with oesophageal cancer. Hutton et al. [41] described that protein intake could be very variable in cancer patients (range 0.2–2.7 g per kg body weight per day) and that a high protein intake did not preclude weight loss. Additionally, Prado et al. [42] showed that 49.0% of cancer patients did not meet protein intake requirements

Our findings suggest a need to explore the impact of estimating protein needs based on body composition, since current weight-adjusted recommendations are inappropriate for healthy subjects or clinical populations, and do not take into account inter-individual variability in protein intakes. There is a need to distinguish absolute or relative protein intake for specific goals of weight maintenance, loss or gain to avoid unfavorable changes in body composition (i.e., loss of LM), which could be achieved by the direct assessment of body composition and REE in clinical practice. Absolute protein intake seems to be more important than the proportion of protein to energy, which changes with the recommended energy needs.

## 5. Conclusions 

Recommendations of protein needs based on LM are promising in different healthy or clinical conditions and should be explored further. In conclusion, body weight-based recommendations are inadequate to classify the differences in protein needs of healthy subjects or clinical populations. 

## Figures and Tables

**Table 1:** Characteristics of healthy subjects, cirrhotic and cancer patients.

**Healthy Subjects**			
	All (*n* = 574)	Females (*n* = 319)	Males (*n* = 255)
Age	41.4 ± 15.2	39.9 ± 15.2 *	43.2 ± 15.2
Height (cm)	172.8 ± 8.5	167.8 ± 6.5 *	179.0 ± 6.3
Weight (kg)	82.2 ± 18.7	78.1 ± 19.8 *	87.3 ± 15.8
BMI (kg/m^2^)	27.4 ± 5.6	27.6 ± 6.2	27.2 ± 4.5
Lean mass_DXA_ (kg) ^1^	54.6 ± 11.9	46.6 ± 7.6 *	64.6 ± 8.2
Muscle mass_MRI_ (kg)	26.1 ± 6.8	21.4 ± 3.8 *	31.7 ± 5.1
ASMI_DXA_ (kg/m^2^)	8.2 ± 1.6	7.4 ± 1.2 *	9.3 ± 1.3
FMI_DXA_ (kg/m^2^)	8.0 ± 15.2	9.8 ± 4.2 *	9.3 ± 1.3
Non Sarcopenic	81.0%	80.9%	81.2%
Sarcopenic	19.0%	19.1%	18.8%
**Cirrhotic Patients**			
	All (*n* = 403)	Females (*n* = 193)	Males (*n* = 210)
Age	44.7 ± 12.3	44.5 ± 12.9	44.9 ± 11.8
Height (cm)	170.5 ± 9.1	164.6 ± 6.7 *	175.9 ± 7.5
Body Weight (kg)	67.5 ± 14.3	59.9 ± 11.0 *	74.4 ± 13.5
BMI (kg/m^2^)	23.1 ± 3.9	22.1 ± 3.6 *	24.0 ± 3.9
Lean mass_bia_ (kg) ^1^	50.7 ± 10.5	43.1 ± 5.6 *	57.7 ± 9.1
Total body protein_TBK_ (kg)	7.5 ± 2.3	6.5 ± 1.5 *	8.8 ± 2.4
FFMI_BIA_ (kg/m^2^)	17.3 ± 2.4	15.9 ± 1.6 *	18.6 ± 2.3
FMI_BIA_ (kg/m^2^)	5.8 ± 2.7	6.2 ± 2.8 *	5.4 ± 2.5
Non Sarcopenic	70.7%	69.4%	71.9%
Sarcopenic	29.3%	30.6%	28.1%
**Cancer Patients**			
	All (*n* = 547)	Females (*n* = 157)	Males (*n* = 390)
Age	61.3 ± 8.2	62.8 ± 7.8 *	60.8 ± 8.4
Height (cm)	168.7 ± 9.6	158.4 ± 6.8 *	172.8 ± 7.1
Weight (kg)	70.8 ± 14.0	63.6 ± 11.4 *	73.7 ± 13.9
BMI (kg/m^2^)	24.8 ± 3.9	25.3 ± 4.1 *	24.6 ± 3.8
Lean mass_CT_ (kg) ^1^	48.6 ± 9.6	38.2 ± 4.9	52.8 ± 7.7
ASMI_CT_ (kg/m^2^)	6.6 ± 1.0	5.9 ± 0.8 *	6.9 ± 0.9
FMI_CT_ (kg/m^2^) ^2^	7.9 ± 2.3	9.2 ± 2.4 *	7.3 ± 2.1
Non Sarcopenic	53.4%	77.1%	43.8%
Sarcopenic	46.6%	22.9%	56.2%

Data are presented as means ± SD or percent; * significant differences between females and males (*t*-test): *p* < 0.05; ASMI = appendicular skeletal muscle mass index; BMI = body mass index; FFMI = fat free mass index; FMI = fat mass index; BIA = Bioelectrical Impedance Analysis; DXA = Dual-energy X-ray absorptiometry; MRI = Magnetic resonance imaging; TBK = Total body potassium; CT = Computer tomography; ^1^ Lean mass = Lean soft tissue_DXA_ in healthy subjects; Fat free mass_BIA_ in cirrhotic patients and Lean soft tissue_CT_ in cancer patients; ^2^ *n* = 531 total; 153 females; 378 males.

## Supplementary Materials

The following are available online at www.mdpi.com/2072-6643/9/1/23/s1, Table S1: Measured (by indirect calorimetry) and calculated (according to Harris and Benedict [1]) resting energy expenditure (REE) in kcal per day in healthy subjects (*n* = 574) and cirrhotic patients (*n* = 403).

## Abbreviations

ASMI	Appendicular Skeletal Muscle Mass Index
BIA	Bioelectrical impedance
BMI	Body mass index
CR	Caloric restriction
CT	Computer Tomography
DXA	Dual energy X-ray absorptiometry
FFM	Fat free mass
FFMI	Fat free mass index
FM	Fat mass
FMI	Fat mass index
LM	Lean mass
LST	Lean soft tissue
MM	Muscle mass
MRI	Magnetic resonance imaging
OF	Overfeeding
REE	Resting energy expenditure
RF	Refeeding
TBK	Total body potassium
TBPro_TBK_	Total body protein
TEE	Total energy expenditure
