# Supplementary material for: Inadequacy of Body Weight-Based Recommendations for Individual Protein Intake—Lessons from Body Composition Analysis
Source: Nutrients. 2016 Dec 31;9(1):23. doi: 10.3390/nu9010023 (PMC5295067; doi:10.3390/nu9010023)
Supplement: Supplementary file 1 [file nutrients-09-00023-s001.pdf]

Supplementary Materials: Inadequacy of Body Weight-Based Recommendations for Individual Protein Intake—Lessons from Body Composition Analysis

Corinna Geisler, Carla M. Prado and Manfred J. Müller

Table S1. Measured (by indirect calorimetry) and calculated (according to Harris and Benedict [1]) resting energy expenditure (REE) in kcal per day in healthy subjects (n = 574) and cirrhotic patients (n = 403) [1].

	Healthy Subjects		Cirrhotic Patients	
REE (kcal/day)	Measured	Calculated	Measured	Calculated
All	1671.9 ± 325.2 *	2062.7 ± 471.7	1697.1 ± 206.4 *	1488.8 ± 244.2
Females	1521.9 ± 261.2 *	1965.9 ± 499.9	1560.4 ± 109.7 *	1311.9 ± 122.5
Males	1864.2 ± 297.1 *	2186.8 ± 400.9	1822.6 ± 194.5 *	1651.3 ± 213.4
Non Sarcopenic	1687.9 ± 332.4 *	2065.6 ± 482.0	1754.9 ± 125.4 *	1530.9 ± 250.5
Sarcopenic	1600.1 ± 281.4 *	2049.8 ± 424.6	1557.4 ± 194.7 *	1387.2 ± 194.7

[1] Data are presented means ± SD; * significant differences between measured and calculated resting energy expenditure (paired t-test): $p < 0.05$; REE: resting energy expenditure.

Reference

1. Harris, J.A.; Benedict, F.G. *A Biometric Study of Basal Metabolism in Man*; Carnegie Institution of Washington: Washington, DC, USA, 1919.
